# GSK3 Is a Central Player in Retinal Degenerative Diseases but a Challenging Therapeutic Target

**DOI:** 10.3390/cells11182898

**Published:** 2022-09-16

**Authors:** Catherine Hottin, Muriel Perron, Jérôme E. Roger

**Affiliations:** Paris-Saclay Institute of Neuroscience, CERTO-Retina France, CNRS, Université Paris-Saclay, 91400 Saclay, France

**Keywords:** GSK3, retina, retinal degenerative diseases, degeneration, neuroprotection, photoreceptors, ganglion cells, signaling pathways

## Abstract

Glycogen synthase kinase 3 (GSK3) is a key regulator of many cellular signaling processes and performs a wide range of biological functions in the nervous system. Due to its central role in numerous cellular processes involved in cell degeneration, a rising number of studies have highlighted the interest in developing therapeutics targeting GSK3 to treat neurodegenerative diseases. Although recent works strongly suggest that inhibiting GSK3 might also be a promising therapeutic approach for retinal degenerative diseases, its full potential is still under-evaluated. In this review, we summarize the literature on the role of GSK3 on the main cellular functions reported as deregulated during retinal degeneration, such as glucose homeostasis which is critical for photoreceptor survival, or oxidative stress, a major component of retinal degeneration. We also discuss the interest in targeting GSK3 for its beneficial effects on inflammation, for reducing neovascularization that occurs in some retinal dystrophies, or for cell-based therapy by enhancing Müller glia cell proliferation in diseased retina. Together, although GSK3 inhibitors hold promise as therapeutic agents, we highlight the complexity of targeting such a multitasked kinase and the need to increase our knowledge of the impact of reducing GSK3 activity on these multiple cellular pathways and biological processes.

## 1. Introduction

The ubiquitously expressed Glycogen synthase kinase 3 (GSK3) has a central role in the cells, being at the crossroad of multiple signaling pathways. It is a phosphorylation target of numerous kinases, which fine-tunes its own kinase activity to modulate multiple downstream targets. As such, GSK3 is a key regulator of many cellular processes and a wide range of biological functions, including the development and maintenance of homeostasis of the central nervous system (CNS) [1]. It participates in neurogenesis [2], neuronal migration and polarization [3,4], microtubule dynamics [5], growth and axon guidance [6,7], and synaptic plasticity [8]. It is therefore not surprising that numerous studies identified GSK3 deregulation as implicated in a large range of CNS disorders, such as Alzheimer’s (AD) and Parkinson’s diseases (PD) [9,10,11]. For instance, increased activity of GSK3 was reported in the brain of AD patients [12,13], and associated to neuronal loss induced by intrinsic apoptosis [14,15]. GSK3 indeed phosphorylates proteins implicated in the development and progression of AD [16,17,18]. Similar implication of GSK3 dysfunction was also reported in PD progression [13,19,20,21,22,23]. GSK3 inhibition therefore presents a great therapeutic option and has already shown beneficial effects in both AD [24,25] and PD [26,27]. GSK3 dysregulation is also observed in other cognitive disorders such as depression, bipolarity, and schizophrenia, which involve very often the same mechanism of phosphorylation and stabilization of toxic complexes [28]. Thus, GSK3 is a therapeutic target of interest for treating CNS related diseases, not only AD [24,25,29,30,31,32,33] and PD [26,27,34], but also Schizophrenia [35], bipolar disorder [36], or depression [37,38]. The therapeutic interest in targeting GSK3 for various brain disorders has been extensively reviewed [39,40,41]. During the last decade, multiple studies also reported GSK3 crucial role in the development and maintenance of the retina [42,43]. Furthermore, it was recently proposed that GSK3 is a key player in retinal neuronal death in various retinal diseases. In this review, we decided to focus on the current knowledge and remaining questions about the mechanisms underlying GSK3 implication in the pathogenesis of retinal dystrophies and to highlight the therapeutic potential of targeting GSK3 to treat these diseases.

## 2. GSK3 Isoenzymes

GSK3 is a highly conserved serine/threonine kinase encoded by two different genes, *Gsk3α* and *Gsk3β* [44,45,46]. GSK3α and GSK3β proteins share 85% amino acid identity, and up to 98% in their kinase domain [46]. GSK3α is the longer isozyme, due to a glycine-rich extension at the N-terminus. GSK3β, also named GSK3β1, has a splicing isoform GSK3β2. This isoform is 13 amino acids longer version of GSK3β, found in rodents [47] and humans [48,49]. Expression of GSK3 is ubiquitous and the highest levels are found in the brain [46], where GSK3β is enriched compared to GSK3α [50]. GSK3β2 is less abundant than its shorter isoform, with the highest expression level found in neurons during development [49]. In this review, unless it is specified, GSK3 will refer to both isozymes indistinctly.

GSK3 activity is regulated through phosphorylation [51,52]. Constitutive phosphorylation in the binding site on tyrosine residues, Y279 for GSK3α, and Y216 for GSK3β, increases the kinase activity [53]. In contrast, phosphorylation of serine residues, S21 for GSK3α, and S9 for GSK3β, leads to the inactivation of the kinase activity [54,55].

GSK3 has been first discovered as the kinase regulating the insulin pathway by phosphorylation of Glycogen Synthase (GS) [56,57,58]. Since then, over 100 substrates have been reported [59], and a lot more predicted [60]. Phosphorylation of GSK3 targets occurs often on a consensus motif (S/T-XXX-PhosphoS/T), with a 90% preference for primed substrates, phosphorylated by another kinase [61]. This initial phosphorylation helps for the positioning of the substrate into GSK3 binding site to trigger a sequential phosphorylation of every four amino acids from the C-ter to the N-ter [61,62]. Very often, such sequential phosphorylation by GSK3 leads to the recognition of a destruction motif by an E3 ubiquitin ligase, mostly by the beta-transducin repeat containing protein (β-TrCP), which recognition motif is similar to the GSK3 target consensus sequence [63]. Poly-ubiquitination on the motif targets the protein to the Ubiquitin/Proteasome System (UPS) for proteasomal degradation [64]. Therefore, phosphorylation mediated by GSK3 leads, with some exceptions, to the degradation of the substrates.

The two isozymes share a high degree of functional redundancy. However, multiple studies were able to highlight some specific functions using different tools including various transgenic mouse lines. For instance, *Gsk3α^−/−^* mice are viable [65] whereas *Gsk3β^−/−^* mice die at late embryonic stages [66].

GSK3 is a central node of numerous signaling pathways by regulating the stability of their intracellular effector through regulatory mechanisms largely described [67]. Among others, GSK3 regulates Insulin, Wnt, Notch, and Sonic Hedgehog signaling pathways [1,61]. GSK3-dependent regulation of the Wnt pathway occurs through sequential phosphorylation of its main effector, β-catenin, on residues Thr 41, Ser 37, and Ser 33, after a priming phosphorylation on Ser 45 by Casein kinase 1 (CK1). The Wnt signaling pathway positively regulates cell proliferation by induction of cell cycle regulators and cell polarity, and represses cell differentiation [68]. In the absence of Wnt ligand binding on Frizzled receptors (Fzl), GSK3 is part of the “destruction complex”, which sequentially phosphorylates β-catenin leading to its poly-ubiquitination and degradation within the proteasome. Activation of the Wnt canonical pathway involves Wnt ligand interaction with one of the Frizzled receptors and LDL receptor-related protein 5/6 (LRP5/6) followed by the inactivation of GSK3 and recruitment of the destruction complex to the membrane, resulting in β-catenin stabilization, which can in turn translocate into the nucleus to regulate target gene expression, such as Cyclin D1, c-JUN, VEGF [62,69]. However, β-catenin is only one of the targets of GSK3 kinase and we will review thereafter that GSK3 regulates many other targets and cellular pathways critical in the context of retinal degenerative diseases.

## 3. GSK3 Involvement in Cellular Functions Deregulated during Retinal Degeneration

The neural retina is composed of six major neuronal cell types, i.e., rod and cone photoreceptors (PRs), horizontal, bipolar, amacrine, and retinal ganglion cells (RGCs) [70,71], and one glial cell type, the Müller glial cells (MGCs) [72]. PRs are highly metabolic cells, and the renewal of the visual pigments and their outer segment is ensured by the retinal pigment epithelium (RPE). It maintains retinal function through the exchange of metabolites between choroidal capillaries and photoreceptors [73,74]. Blindness is mainly due to retinal degenerative diseases affecting PRs or RGCs. Degeneration of RGCs and their axons forming the optic nerve cause glaucoma, which is the main leading cause of blindness worldwide. Age-related macular degeneration (AMD), a multifactorial degenerative disease, is another leading cause of blindness. The loss of vision is due to an alteration of the RPE and the cone photoreceptors located in the macula, leading to a loss of the central vision [75,76]. The most common inherited PRs degeneration is retinitis pigmentosa (RP). The phenotype is characterized by a primary loss of rod photoreceptors followed by a secondary cone cell death [77]. It is one of the most clinically and genetically heterogenous diseases with over 100 causing genes identified making it challenging to develop effective treatments. Other retinal degenerative diseases can arise from another disease. For instance, diabetic retinopathy (DR), leading to retinal neovascularization associated with macular edema and retinal degeneration, is a complication of Type-2 diabetes [78]. We will address below the involvement of GSK3 in different cellular functions involved in retinal degeneration, including apoptosis, glucose metabolism, and oxidative stress, offering multiple entry points for therapy downstream GSK3.

### 3.1. GSK3 and Cell Apoptosis

Although GSK3 first identified function was linked to glucose metabolism, this kinase is presently known to be a key regulator of cell apoptosis, making it a prime target for degenerative diseases [79]. Regarding the underlying mechanisms, it was shown that GSK3β, downstream of PI3K signaling, promotes cell death under certain circumstances, by inhibiting pro-survival transcription factors while promoting p53-mediated apoptosis. The use of small-molecule GSK3 inhibitors SB-216763 and SB-415286 has also supported a pro-apoptotic role for GSK3 in primary neuronal cells [80]. Surprisingly however, GSK3 has also been found to function as a pro-survival enzyme. These apparent paradoxical anti- and pro-apoptotic roles of GSK3 have been extensively described and are now known to be due to opposite GSK3 functions in the regulation of the two major apoptotic signaling pathways [79,81,82]. Indeed, GSK3 has a pro-apoptotic role through the mitochondrial intrinsic apoptotic pathway by acting on targets contributing to the alteration of the mitochondria and the release of cytochrome c [83,84,85,86]. In contrast, GSK3 has an anti-apoptotic role through the extrinsic apoptotic pathway, which is mitochondria independent, by preventing death-inducing signaling complex (DISC) formation and subsequent activation of caspases [86]. In this context, pharmacological inhibition of GSK3 can protect neural cells from intrinsic apoptosis signaling [87] but can at the same time potentiate their death by the extrinsic pathway [80,88]. Bearing in mind these opposite functions of GSK3 on two regulatory mechanisms of cell apoptosis is obviously crucial for the rational use of GSK3 inhibitors for therapeutic interventions. In addition, a better knowledge of the different cell death pathways involved in the different neurodegenerative diseases is a prerequisite to identify disease candidates for GSK3-mediated therapies.

In the retina, the direct role of GSK3 in regulating apoptotic pathways during retinal degeneration is still poorly understood. GSK3 expression levels do not change upon retinal degeneration, but GSK3 phosphorylation, and thus its activity, is modulated. For instance, the phosphorylation of GSK3β^Ser9^ is decreased in rat retinas by N-Methyl-N-nitrosourea (MNU) treatment, a well-established paradigm for inducible photoreceptor cell death [89], suggesting an increase in GSK3β activation [90]. In diabetic mice with DR, a positive correlation between GSK3 activation and retinal neuron apoptosis has also been reported [91,92]. Similarly, the levels of phosphorylated Ser21/9 in GSK3α/β are lower in a serum-deprived RGC cell line model [93]. This increased activity likely contributes to cell death progression since increased levels of phosphorylated Ser21/9 in GSK-3α/β following lithium chloride (LiCl) treatment was associated with a slower rate of apoptosis and increased mitochondrial membrane potential [92]. In the context of glaucoma, it has been suggested that GSK3β facilitates RGC death via the upregulation of dynamin-related protein 1 (DRP1) and mitochondrial fission [94]. Therefore, considering the pro-apoptotic function of GSK3, several inhibitors have been tested in animal models of retinal diseases to reduce photoreceptor or RGC death [40]. For instance, GSK3 inhibition with LiCl protects rat retinas against MNU-induced degeneration [90]. Similarly, GSK3 inhibition using VP3.15 has a beneficial effect against photoreceptors degeneration, in *rd10* mice, a commonly used model of RP [95], both ex vivo [96] and in vivo [97]. Of note, in this *rd10* mouse model, levels of GSK3β^Ser9^ in the retina were shown to be increased, suggesting reduced GSK3 activity [95,97]. Whether this inhibitory phosphorylation represents an intrinsic neuroprotective response to the degeneration remains to be investigated. Furthermore, the mechanisms underlying the apparent opposite impact of different models of degeneration on GSK3 activation are so far unknown.

Although reducing GSK3 activity appears beneficial for neuroprotection in models of retinal degeneration [97,98], the complete deletion of *Gsk3α* and *Gsk3β*, specifically in retinal progenitors, leads to massive cell death by apoptosis [43]. Remarkably, only one allele of *Gsk3α* or *Gsk3β* is sufficient to fully differentiate a functional retina demonstrating their overall functional redundancy in the retina [43]. Interestingly, this genetic context of a single wild-type allele of either *Gsk3α* or *Gsk3β* leads to an increased number of displaced RGCs in the inner nuclear layer, suggesting that GSK3 contributes to the production of specific cell types. Regarding cell survival, only one functional allele of *Gsk3β* leads to reduced cell death in mouse models of retinal degeneration, similar to drug-induced GSK3 inhibition, offering a powerful genetic model to identify specific deregulated target genes (our unpublished data).

To sum up, although the underlying molecular mechanisms still remain to be investigated, GSK3 in the retina contributes to the degenerative process while reducing its activity is associated with reduced cell death.

### 3.2. GSK3 and Retinal Glucose Homeostasis

GSK3 is long known to be a key regulator of glycogen content synthesis in response to insulin and therefore of glucose metabolism. It was indeed originally named for its ability to phosphorylate and inhibit glycogen synthase (GS), the enzyme that catalyzes the conversion of glucose into glycogen [56]. In the retina, the presence of glycogen was demonstrated both in neurons [99] and in MGCs [100]. It has been suggested that glycogen could be used as an immediate accessible energy reserve in the retina [101]. Both GS and GSK3β were found expressed in MGCs and in the inner segments of the PRs [43,98]. As mentioned above, PRs are the most active metabolic cells in the retina. One of the main reasons is the necessity to continuously renew their outer segments. The expression of both GS and GSK3β in these highly metabolic cells suggests that the handling of glycogen could be regulated within photoreceptors themselves and that GSK3 would be a key regulator of this process. Inhibiting GSK3 as a therapeutic strategy may thus potentially impair glucose homeostasis. This could be an important issue since there is evidence that glycogen accumulation in neurons leads to neurodegeneration [102]. On the other hand, it has been suggested that the hypoglycemic conditions observed in diabetes could compromise retinal neuronal survival [103]. Therefore, it remains to be tested whether drug-inhibition of GSK3 in various genetic contexts could impact cell survival via its function on glucose metabolism.

**Figure 1:**
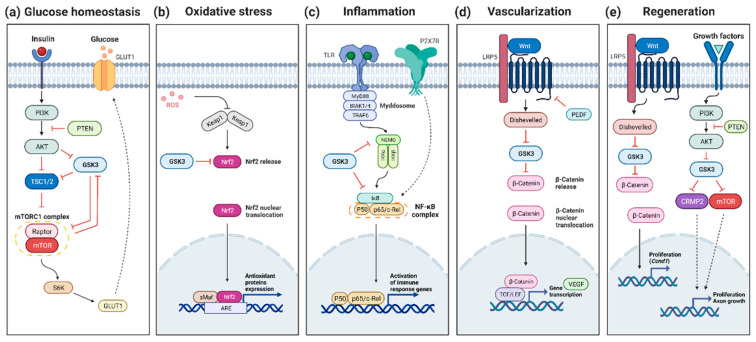
GSK3 regulatory function in different signaling pathways. (**a**) GSK3 regulates glucose homeostasis through the inhibition of TSC1/2 and Raptor/mTOR complex. (**b**) Nrf2 release, mediating the oxidative stress response, is inhibited by GSK3. (**c**) GSK3 regulates inflammation through the phosphorylation of NF-κB and upstream effectors. (**d**) Wnt signaling pathway activation induces VEGF expression and as such positively regulates vascularization. (**e**) Release of GSK3 inhibition following Wnt and growth factors addition enhances regeneration through *Ccnd1* induction and mTOR/CRMP2 signaling, respectively. Created with BioRender.com.

In RP, several hypotheses have been proposed to explain the secondary cone cell death occurring after the loss of rods. Several data converge on the idea that cones die, at least in part, from starvation, and that a plausible therapeutic avenue for neuroprotection could be achieved through the stimulation of glucose metabolism [104,105,106]. The target of rapamycin (mTOR) is a key regulator of genes involved in glycolysis [107]. Systemic injection of insulin stimulating the mTOR pathway was shown to prolong cone photoreceptor survival for a few weeks [104]. Similarly, constitutive activation of the mTOR pathway revealed to be efficient for promoting long-term cone survival in the retina in two mouse models of RP, a fast and a slow-progressing models, *rd1* and *rhodopsin*-KO, respectively [108]. In contrast, depletion of insulin was found to accelerate cone cell death [104]. GSK3 has been shown to inhibit the mTOR pathways at different levels although such regulation has not yet been demonstrated in the retina [109,110,111] (Figure 1a). In this context, and in contrast to the classical approach aimed at inhibiting GSK3 to prevent cell death, sustained activation of GSK3 might be beneficial for late stages of RP, to preserve the starving cones by promoting mTOR signaling.

### 3.3. GSK3 and Retinal Oxidative Stress

Oxidative stress is an important feature in neurodegenerative diseases. It results from reactive oxygen (ROS) and nitrogen species production via the NADPH oxidase complex and the mitochondria during ATP production in the respiratory chain [112]. During aging, mitochondria can progressively dysfunction with an increased production of ROS [113,114]. Any minor changes in oxidative stress signaling as well as the increase of ROS levels can trigger retinal degeneration. In diabetic mice with DR, the increased activation of GSK3 was shown to lead to RGCs degeneration via increased mitochondrial oxidative stress [91]. In humans, increased oxidative stress has been demonstrated in AMD [115], DR [116], and RP [117,118]. Anti-oxidant treatments to lower ROS levels are thus appealing therapeutic approaches [119].

The nuclear factor erythroid-2-related factor 2 (NRF2) has recently emerged as a factor of interest in retinal diseases in the context of oxidative stress, and as regulated by GSK3 [120,121] (Figure 1b). NRF2 is expressed by all types of retinal cells [122,123]. Under high ROS levels, NRF2 is upregulated and helps neutralize the oxidative damage by inducing transcription of antioxidants proteins [124]. Among its negative regulators, GSK3 was shown to phosphorylate NRF2 on the Neh6 domain for subsequent degradation by the UPS [125]. High glucose concentrations in Müller cell cultures also lead to NRF2 inhibition, associated with increased oxidative stress [126]. Such enhanced ROS levels in cells exposed to hyperglycemic culture conditions can be reduced by inhibiting REDD1, a stress response protein. In diabetic mouse retinas, it was shown that REDD1 induces NRF2 degradation via GSK3-dependent phosphorylation. In this mouse model, the pharmacological inhibition of GSK3β, via VP3.15 administration, increases NRF2 activity and prevents the diabetes-induced increase of ROS [127,128]. Moreover, the oxidative damages observed in aging RPE have been linked to NRF2 signaling deregulation [129]. GSK3β pharmacological inhibition, with SB216763, restores NRF2 levels after an oxidative stress challenge and protects against oxidant-induced cell death [130]. Consequently, this supports the potential therapeutic benefit of increasing NRF2 expression through GSK3 inhibition to treat retinal dystrophies associated with high oxidative stress levels. Along this line, some interesting antioxidants under study are the Polo-like kinase 2 (PLK2) and Cannabidiol (CBD). It was shown on cultured RGCs that PLK2 inhibits GSK3β resulting in increased NRF2 signaling, hence in neuroprotection against stress-induction. In vivo activation of cannabinoid receptors in mouse brains resulted in increased GSK3 inhibition [131]. The antioxidant action of CBD is more likely mediated by NRF2 [132]. Importantly, promoting NRF2 activity through GSK3 inhibition already showed promising effects in AD [133,134,135] and PD [136,137], and therefore pave the way to potential treatments for oxidative stress-related retinal diseases.

## 4. GSK3 and Retinal Inflammation

Retinal inflammation is well known to contribute to the pathogenesis of retinal diseases, such as AMD, DR, or RP [138,139,140,141]. Inflammation is one of the important functions that are regulated by GSK3 since it is well established that GSK3 acts as a modulator of inflammatory components [38,142,143]. For instance, GSK3 negatively regulates anti-inflammatory cytokine production such as IL-2, IL-10, IL-22, or IL-33 [144]. Conversely, this kinase acts as a positive regulator of pro-inflammatory cytokines and chemokines, such as TNF-α, interleukin (IL-)1β, IL-6, IL-17, IL-18, IL-23, IL-12, IFN-γ, IL-8, C-C motif chemokine ligand (CCL) 2, 3, 4, and 12, C-X-C motif chemokine ligand (CXCL) 1, 2, 5, and 10 [144]. Together, this raised the possibility that inhibitors of GSK3 may prove to be beneficial for inflammatory conditions. One signaling molecule of interest targeted by GSK3 is NFκB, a pivotal mediator of inflammatory responses that has long been proposed as a potential target for the therapy of inflammatory diseases [145,146] (Figure 1c). It was shown that NF-κB is activated in *rd* mice and light-induced retinal degeneration [147,148] Interestingly, GSK3β was shown to facilitate NF-κB transactivation by TNF-α since GSK3β deficient mouse embryonic fibroblasts exhibit defective NF-κB activation in response to TNFα [66]. Mechanistically, direct phosphorylation of NF-κB subunits p65 by GSK3 was reported in hepatocytes [149]. Along the same line, GSK3 inhibition in microglial cells decreases LPS-induced inflammation through the decrease of the activation of p65 [150]. GSK3 also activates non-canonical NF-κB signaling through the phosphorylation of p100, an inhibitor of NF-κB, targeting it to the proteasome [151]. Moreover, the increased β-catenin levels following GSK3 inactivation may further enhance NF-κB inhibition, since β-catenin was shown to inhibit NF-κB activity through physical interaction [152]. It is however noteworthy that GSK3 could differentially regulate NF-κB activity depending on the physiological state of the cell. Indeed, although it is required for the activation of NF-κB in response to cytokine stimulation [153], it may inhibit NF-κB in resting cultured cells [153,154,155]. Moreover, NF-κB can exhibit anti-apoptotic effects. Indeed, mice with inactivated GSK3β die from hepatocyte apoptosis during development due to a defect in NF-κB activation [66]. Finally, although GSK3 inhibition in different models predominantly contributes to the amelioration of inflammation, it may alternatively lead to the opposite effect as it could also prohibit the termination of inflammation [144]. As a whole, it is clear that more knowledge is needed to evaluate the net outcome of GSK3 inhibitors on NF-κB activity and on neuroinflammation in general in different models of retinal degeneration.

Another interesting factor linked to inflammation and GSK3 is the P2X7 receptor (P2X7-R) which is known to promote chronic neuroinflammation and neurodegenerative brain diseases [156]. In AD mouse models, P2X7-R inhibition was shown to have a protective effect through GSK3 inhibition [157,158]. P2X7-R is expressed in the retina and the RPE, and during retinal degeneration its expression increases [159]. P2X7-R induces the expression of inflammatory factors in the retina [160,161]. Interestingly, the pharmacological inhibition of P2X7-R prevents the increased inflammation and neovascularization induced by oxidative stress in the mouse eye in vivo [162]. It would be interesting to know whether some of these effects on inflammation and neuroprotection involve GSK3 inhibition.

## 5. GSK3 and Retinal Vascularization

Retinal neovascularization is observed in some retinal degenerative diseases, such as DR or wet AMD, where there is a disruption of the blood-retinal barrier (BRB). This phenotype is correlated with the increased expression of Vascular Endothelial Growth Factor (VEGF), a target gene of Wnt signaling [163,164]. A commonly used treatment strategy for wet-AMD and DR relies on reducing angiogenesis via anti-VEGF agents [165,166,167,168,169,170,171].

Wnt signaling activation promotes retinal vascularization [172,173,174], BRB development, and maintenance [175]. Accordingly, the reduction of Wnt signaling by the loss of LRP5, a canonical Wnt co-receptor, suppresses pathologic neovessel formation in a mouse model of oxygen-induced retinopathy [176]. Even if the loss of LRP5 negatively regulates retinal neovascularization in development and adulthood, there are still some vessels formed, with great disorganization and blood leakage [177]. Acting on downstream effectors of Wnt signaling could be another strategy to prevent neovascularization. In this pathological context, promoting GSK3 activity might thus be an interesting alternative approach to inhibit Wnt signaling and subsequently diminish VEGF expression (Figure 1d).

In contrast to the occurrence of neovascularization in some retinal dystrophies, other eye diseases are characterized by the poor formation of intraocular vasculature, such as the familial exudative vitreoretinopathy (FEVR) [178]. Consistent with the importance of Wnt signaling in retinal vascular development, Wnt inhibition in *Lrp5^−/−^* mice produces eye vascular pathologies that model FEVR in humans [179]. Importantly, inhibition of GSK3 by LiCl treatment in this mouse model was shown to rescue defective retinal vasculature through restoring Wnt signaling, providing a potential treatment approach for FEVR [179].

## 6. GSK3 and Retinal Regeneration

Cell-based therapy is an appealing approach in late-stage retinal degeneration when most cells are already dead. One approach relies on the stimulation of endogenous repair processes. Some species have a high regenerative capacity, such as zebrafish or *Xenopus*, in which MGCs exhibit stemness properties. After retinal damage, dormant Müller cells can exit quiescence, proliferate and differentiate into different retinal cell types [180,181,182]. In contrast with fish and amphibians, these stemness and neurogenic capacities are highly limited in the mammalian retina [183,184]. The goal is to identify cellular pathways able in mammals to trigger Müller glia reprogramming and differentiation of Müller-derived progenitors under pathological conditions.

Several results gathered in different animal models converge on the idea that regeneration of retinal neurons can be promoted by applying GSK3 inhibitors to the retina. Zebrafish regenerative capacity observed after retinal damage is mediated by Ascl1 [185], which induces the Wnt signaling pathway [186]. Remarkably, in zebrafish undamaged retinas, Wnt activation, via GSK3 inhibition with LiCl, is sufficient to stimulate MGCs proliferation and induce retinal regeneration [186]. In the chick retina, which has a low regenerative potential, GSK3 drug-inhibition associated with FGF2 treatment promotes MGCs proliferation and dedifferentiation [187]. In the rat retina, although some MGCs are stimulated to proliferate and produce retinal cells in retinal explants, this remains very limited [188]. Activation of Wnt signaling, either by Wnt3a treatments or using SB216763 or AR-A014418 as inhibitors of GSK3β, was shown to promote the proliferation of Müller glia-derived retinal progenitors and neural regeneration in the wild-type retina [189] (Figure 1e). However, in *rd* mice, similar approaches induce MGC proliferation only at P12 but not at a later stage suggesting that the retinal microenvironment variation under pathological conditions might contribute to the lack of retinal repair observed in mammals [189]. Along the same line, the proliferative response of MGCs in mouse retinal explants following the addition of the GSK3 inhibitor Chir99021 varies between mouse strains, highlighting the importance of the genetic background [190]. Nevertheless, altogether these data in different models and species suggest that targeting GSK3 for retinal cell regeneration deserves further attention.

Given the critical function of GSK3 in regulating axon growth, modulation of GSK3 activity may also represent an interesting strategy to trigger axon regeneration following injuries [7,191,192,193,194]. Interestingly enough, antagonist effects were observed on GSK3 inhibition and axon growth. GSK3 activation promotes peripheral nerve axon growth, whereas GSK3 inhibition promotes CNS axon growth [195]. Lack of phosphatase and tensin homolog expression (*Pten^−/−^)* is neuroprotective and enhances RGC axon regeneration. Indeed, in this model, RGC axonal regeneration in mature neurons is promoted by activation of mTOR [196]. Such a mechanism relies essentially on GSK3 inactivation supporting that GSK3 inhibitors could serve as a regenerative stimulus.

Axon regeneration is an appealing approach for preserving the optic nerve in glaucoma [197]. Activation of Wnt signaling through intravitreal administration of Wnt3a after an optic nerve crush (ONC), a classical axon injury paradigm, contributes to axonal regeneration [198]. Consistent with this, GSK3β inhibition also enhances optic nerve regeneration after ONC [195]. This effect is mediated by the Collapsin response mediator protein 2 (CRMP2), a microtubule-binding protein involved in neuronal polarization, migration, and differentiation. Therefore, GSK3/CRMP2 axis is a pathway of interest to treat glaucoma by promoting axon regeneration [196]. Another interesting factor involved in regeneration is mTOR. mTOR positively regulates cell growth, proliferation, and survival, and this is mediated by activation of Wnt pathway, thus GSK3 inhibition [199]. Similarly, mTOR promotes axon regeneration in the CNS through GSK3 inhibition [200]. In the retina, after an ONC and inflammatory stimulation, mTOR signaling enhances optic nerve regeneration as well as RGCs neuroprotection [201]. Thus, inhibiting GSK3 is an appealing strategy for optic nerve regeneration as it should enhance the activity of key downstream effectors, in particular CRMP2 and mTOR.

## 7. Therapeutic Trials Targeting GSK3 in Retinal Degenerative Diseases

Due to the large clinical and genetic heterogeneity of retinal diseases, mutation-agnostic therapeutic approaches based on neuroprotection, are appealing strategies. Some factors were identified as neuroprotective against PRs [202] or RGCs degeneration [203], such as brain derived neurotrophic factor (BDNF), ciliary neurotrophic factor (CNTF), pigment epithelium-derived factors (PEDF), glial cell line-derived factor (GDNF) [204]. Chemical compounds also offer a therapeutic avenue for treating inherited retinal diseases. Given (i) the known implication of GSK3 in brain disorders, (ii) the similarities between brain neurodegenerative conditions and retinal degenerative diseases, and (iii) as reviewed above, the implication of GSK3 in various cellular processes involved in retinal degeneration, lowering GSK3 activity using GSK3 inhibitors in the retina is an appealing potential therapeutic approach.

In humans, oral intake of valproic acid (VPA) in a clinical trial revealed a short-term benefit to patients with RP [205]. VPA is known to inhibit GSK3 [206,207,208]. The observed neural protection of VPA is indeed mediated by GSK3 inhibition [208]. In another study, however, VPA failed to show clinical benefit in autosomal dominant RP patients [209]. One possible explanation comes from a study in different *Xenopus* models of RP, in which VPA was shown to have either beneficial or detrimental effects depending on the disease mechanisms and therefore suggesting that the success or failure of VPA treatment is dependent on the patient genotype [210]. Such variability highlights the need to increase our knowledge of the mechanisms underlying GSK3 inhibition in order to fully leverage its therapeutic value.

Concerning RGC protection, the inhibition of GSK3 with small molecules showed a beneficial effect against cell death in diabetic mice with DR [91,92,211,212], and in the N-methyl-D-aspartate (NMDA) neurotoxicity model of mouse retinal explants [96]. The use of siRNA to knockdown GSK3β after rat optic nerve crush also suggested that GSK3 inhibition is neuroprotective for RGCs [213].

Overall, these studies using GSK3 inhibitors showed some beneficial effects for RP or glaucoma diseases and thus GSK3 represents an interesting therapeutic target option that deserves further investigations.

## 8. Precautions and Advantages of Inhibiting GSK3 or GSK3 Targets as a Therapeutic Strategy for Eye Diseases

Retinal diseases are numerous but biological processes deregulated in each of them are often shared [11]. In this context, factors at the crossroad of multiple pathways offer great potential as therapeutic targets. Among them, GSK3 has been shown deregulated in multiple CNS disorders including retinal diseases and due to its central role in regulating multiple signaling pathways, both kinases and their downstream targets represent targets of choice (Table 1), especially in a nonstop growing aging population. However, several points must be considered with caution when considering GSK3 as a therapeutic target.

At first, it is important to consider the type of disease, its dynamics and its evolution. Indeed, using GSK3 inhibitors to modulate GSK3 activity should be finely regulated, depending on the disease and the disease’s stage. For the case of DR, GSK3 is reported as activated and its inhibition might be beneficial at the early stage of the disease to preserve RGCs [91] but then keeping GSK3 inactivated might promote VEGF expression and angiogenesis through downstream effectors of the Wnt pathway [172,173,174]. Along the same line, in AMD, GSK3 inhibition could be beneficial in the dry form when neovessels are absent, but deleterious in the wet form by promoting retinal neovascularization. One could speculate that the combination of GSK3 inhibitors with other drugs, such as anti-VEGF, could at the same time inhibit angiogenesis and enhance cell survival. Only after such a thorough analysis of GSK3 regulation and its implication in the disease progression can the use of a GSK3 inhibitor be considered. This also raises the question of whether GSK3 inhibition should be transient or chronic. Along the same line, regeneration could be initiated and enhanced by transient inhibition of GSK3 to induce MGC proliferation but might not be required thereafter, to avoid over-proliferation or allow further differentiation. Overall, the time window and the duration of the treatment must be fine-tuned and defined for each disease, for a specific stage of the disease, and/or for a particular cell type.

One more thing to consider is that GSK3 is ubiquitously expressed and presents different functions across the different cell types/tissues. The drug effect might not have the same efficacy whether it is administered in a systemic way or in a tissue-selective manner by local treatment. In contrast with the brain, the eye is easy to access, therefore intravitreal injection is often preferred. As such, it solves one of the issues of the pharmacological treatment targeting GSK3 activity and the ability of these drugs to cross the retinal blood barrier. This type of injection is commonly used in ophthalmology for treating wet forms of AMD by repeated injection of anti-VEGF to reduce the neovascularization occurring in this form and therefore delay photoreceptor degeneration. Therefore, an effort should be put into the research of carriers providing local delivery of the product using intravitreal injection. To achieve such local and long-term delivery of the drugs, liposomes or polymeric nanoparticles could be a solution as a drug carrier [214].

Yet, another point to be resolved is to know if both isozymes should be targeted regarding their high degree of functional redundancy to maximize the effects. Of note, most used inhibitors are targeting GSK3β, but it might also affect GSK3α. Regarding the literature, inhibition of both isozymes might be favorable [41]. Another unresolved issue nowadays is to precisely distinguish the role of each GSK3 isozyme in each pathology, depending on the stage of the disease.

Another strategy could be to target downstream pathways and substrates of GSK3, known to be involved in the pathology and allowing a neuroprotection through specific inhibition or activation. Indeed, due to the broad spectrum of GSK3 cellular targets (over 100 known substrates), its inhibition in the brain by the means of small molecules logically leads to many side effects and as a consequence few GSK3 inhibitors have reached phase 2 clinical trials. Therefore, a better strategy for more effective therapies could instead target specific GSK3 downstream targets. For instance, some promising treatments targeting GSK3-regulated pathways are combining anti-oxidant effects and anti-inflammatory effects, as is the case of flavonoids coming from fruits and vegetables [215,216] or cannabidiol [217]. To illustrate the interest in targeting GSK3 targets, one can cite NRF2 for its antioxidant and anti-inflammatory functions. Several studies demonstrated the therapeutic interest of overexpressing this factor using AAV vectors. NRF2-based gene therapy showed neuroprotective effects against oxidative stress [218]. Interestingly, a ganglion-cell-specific promoter *Mcp-1* was used as it is expressed only in stressed RGCs, avoiding non-cell-specific effects. NRF2 gene therapy showed also neuroprotective effects in a mouse model of AMD using light damage [219]. Another GSK3 target relevant for RGCs preservation is CRMP2. A gene therapy approach in rats based on the expression of a constitutively active form of CRMP2 resulted in RGCs neuroprotection after an optic nerve injury [220]. An additional promising target discussed above is mTOR. Upregulation of mTOR by inhibition of upstream effectors, such as PTEN and TSC2, has a positive role in optic nerve regrowth [221]. Interestingly, in the cases of DR and wet AMD, inhibition of mTOR using drug inhibitors or AAV seems to be a good option to reduce vascularization by preserving endothelial structure [222,223,224]. Altogether, these preclinical results clearly demonstrated the therapeutic interest in targeting GSK3 substrates.

**Table 1:** Overview of GSK3-putative or -demonstrated role under different retinal degenerative context. Abbreviations: AD: Alzheimer’s disease; AMD: age-macular degeneration; BRB: blood-retinal barrier; CBD: cannabidiol; CNS: central nervous system; DR: diabetic retinopathy; FEVR: familial exudative vitreoretinopathy; GS: glycogen synthase; GSK3: glycogen synthase kinase 3; LPS: lipopolysaccharides; MGC: Müller glial cell; MNU: N-methyl-N-nitrosourea; PR: photoreceptor; *rd*: retinal degeneration; RGC: retinal ganglion cell; RP: retinitis pigmentosa; RPE: retinal pigment epithelium.

	Cell Type	Cell Death Model	GSK3	Observed Effects	Ref.
_	PRs	RP (*rd10*)	inactivation	PRs neuroprotection	[96,97]
		MNU	inhibition (lithium)		[90]
	RGCs	RGCs degeneration	activation	RGCs death	[195]
			inhibition	promotes RGCs survival, axon regeneration	[90,195,213]
		DR	activation	mitochondrial oxidative stress increase, RGCs degeneration	[91]
				RGCs degeneration	[211]
	astrocytes			Ang2 increase, astrocytes apoptosis, BRB disruption	[212]
	RGCs, glial cells, astrocytes	DR	inhibition	RGCs, glial cells and astrocytes neuroprotection	[91,92,211,212]
glucose homeostasis		_	activation	GS phosphorylation, prevent glucose to glycogen conversion	[56]
	CNS	_	_	neurodegeneration due to glycogen accumulation	[102]
	PRs	DR	_	hypoglycemia compromise neuronal survival	[103]
		RP	_	mTOR activation preserves cone photoreceptors	[104,108]
		RP	_	insulin depletion accelerates cone death	[104]
oxidative stress	PRs	AMD	_	oxidative stress increase	[115]
		DR	_	oxidative stress increase	[116]
		RP	_	oxidative stress increase	[117,118]
		early DR	activation	NRF2 degradation	[127,128]
			inhibition	NRF2 increased expression, neuroprotection	
	MGCs	high glucose	_	oxidative stress increase, NRF2 decrease	[126]
	RPE	_	inhibition	NRF2 signaling rescue	[130]
	RGCs		inhibition (through PLK2)	cell survival	[225]
		glaucoma	_	CBD neuroprotection	[226]
		DR	_		
			inhibition (through CBD)	NRF2 signaling induction	[131,132]
inflammation	PRs	*rd* mice	_	NF-κB activation	[147]
		light induced	_		[148]
	RGCs	optic nerve crush	_		[227]
	microglial cells	_	inhibition	decrease of LPS-induced inflammation. NF-κB activation, decrease of TNFα secretion	[150]
	CNS	AD	Inhibition	P2X7 inhibition via GSK3, neuroprotection	[157,158]
	eye	oxidative stress	_	P2X7 inhibition prevents inflammation and vascularization	[162]
vascularization	PRs	DR	inactivated (through Wnt signaling)	VEGF production, vascularization	[163,164]
		FEVR	_	Wnt inhibition is a model of FEVR	[179]
		FEVR	inhibition (inhibitor)	rescue of defective retinal vasculature	[179]
regeneration	zebrafish retina	retinal damage	inhibition (through Wnt signaling)	*Ascl1* expression	[185,186]
		retinal damage	inhibition (inhibitor)	sufficient for regeneration	[186]
	chick retina	retinal damage	inhibition + FGF2 treatment	MGCs proliferation and dedifferentiation	[187]
	PRs	*rd* mice	inhibition (through Wnt signaling)	necessary for MG-derived progenitor production, proliferation and reprogramming	[189]
	RGCs		inhibition (through PTEN inhibition)	RGCs neuroprotection, axon regeneration	[196]
		optic nerve crush	inhibition (through Wnt signaling)	CRMP2 signaling induction, axon regeneration	[198]
			inhibition (inhibitor)		[195]
		optic nerve crush + inflammation	_	mTOR signaling increase, optic nerve regeneration, RGCs neuroprotection	[201]
	CNS	_	inhibition	mTOR signaling induction, axon regeneration	[200]

Overall, targeting GSK3 activity is challenging as a therapeutic approach but shows high potential. The challenge would be to target specific cell types for each disease and to determine whether the activation or inactivation should be transient or chronic. In this context, a better understanding of GSK3 function in different retinal diseases and identification of their targets will certainly help in developing new therapeutic approaches.

## Data Availability

Not applicable.
